# Investigating Serious Games That Incorporate Medication Use for Patients: Systematic Literature Review

**DOI:** 10.2196/16096

**Published:** 2020-04-29

**Authors:** Olufunmilola Abraham, Sarah LeMay, Sarah Bittner, Tanvee Thakur, Haley Stafford, Randall Brown

**Affiliations:** 1 Social and Administrative Sciences Division School of Pharmacy University of Wisconsin–Madison Madison, WI United States; 2 Department of Family Medicine and Community Health School of Medicine and Public Health University of Wisconsin–Madison Madison, WI United States

**Keywords:** games, medication adherence, patient safety, video games, systematic review

## Abstract

**Background:**

The United States spends more than US $100 billion annually on the impact of medication misuse. Serious games are effective and innovative digital tools for educating patients about positive health behaviors. There are limited systematic reviews that examine the prevalence of serious games that incorporate medication use.

**Objective:**

This systematic review aimed to identify (1) serious games intended to educate patients about medication adherence, education, and safety; (2) types of theoretical frameworks used to develop serious games for medication use; and (3) sampling frames for evaluating serious games on medication use.

**Methods:**

PubMed, Scopus, and Web of Science databases were searched for literature about medication-based serious games for patients. Preferred Reporting Items for Systematic Reviews and Meta-Analyses (PRISMA) guidelines were followed for article selection.

**Results:**

Using PRISMA guidelines, 953 publications and 749 unique titles were identified from PubMed, Scopus, and Web of Science. A total of 16 studies featuring 12 unique serious games were included with components of medication adherence, education, and safety, published from 2003 to 2019. Of the 12 games included, eight serious games were tested in adolescents, three games were tested in young adults, and one game was tested in adults. Most studies (n=11) used small sample sizes to test the usability of serious games. Theoretical frameworks identified in the 12 serious games included information, motivation, and behavior theory; social cognitive theory; precede-proceed model; middle-range theory of chronic illness; adult learning theory; experiential learning theory; and the theory of reasoned action. Existing reviews explore serious games focused on the management of specific disease states, such as HIV, diabetes, and asthma, and on the positive impact of serious game education in each respective disease state. Although other reviews target broad topics such as health care gamification and serious games to educate health care workers, no reviews focus solely on medication use. Serious games were mainly focused on improving adherence, whereas medication safety was not widely explored. Little is known about the efficacy and usability of medication-focused serious games often because of small and nonrepresentative sample sizes, which limit the generalizability of existing studies.

**Conclusions:**

Limited studies exist on serious games for health that incorporate medication use. The findings from these studies focus on developing and testing serious games that teach patients about medication use and safety. Many of these studies do not apply a theoretical framework in the design and assessment of these games. In the future, serious game effectiveness could be improved by increasing study sample size and diversity of study participants, so that the results are generalizable to broader populations. Serious games should describe the extent of theoretical framework incorporated into game design and evaluate success by testing the player’s retention of learning objectives.

## Introduction

### Background

An estimated 117 million Americans currently live with one or more chronic conditions, many of which require medication management [1]. Using many medications for chronic conditions is accompanied with a high risk of medication errors, insufficient knowledge about appropriate use, and inadvertent adverse drug events. In the United States, a leading reason for injuries and death is because of the estimated 1.5 million medication errors [2]. Common mistakes made by patients or caregivers outside of the hospital include taking a medication twice by accident, an incorrect dose, or the wrong medication [2].

A common barrier to chronic disease management for many patients is medication adherence. Approximately 50% of patients do not take prescribed medication appropriately and consistently [3]. Medication adherence or taking medications correctly is generally defined as the extent to which patients take medication as prescribed by their doctors [4]. Patients, health care providers, and hospital systems would benefit immensely from helping patients use medication correctly, consistently, and safely. The health care system would benefit from gaining the estimated US $100 to US $300 billion every year because of nonadherence alone [5]. Patient knowledge on safe medication practices is critical in preventing unnecessary patient harm. For example, recent reports from Poison Control state that approximately 60,000 children were sent to the emergency room every year because of taking medications without adult supervision [6]. These findings show opportunities for patient education on safe medication use, storage, and disposal, particularly for young people and their family caregivers.

Technology has a significant impact on education and health behavior reinforcement both in patients and providers [7]. Devices such as mobile phones relay information, reinforce norms, and influence behaviors such as medication adherence [7]. The appeal of technology, particularly gaming, makes serious games an ideal approach to portray medication information [8]. Technology-based *serious games* are a novel method of delivering interactive health behavior education through skill-building exercises [9,10]. Serious games are digital tools that offer engagement activities through a responsive narrative to educate participants through role-play and practicing skills. Unlike traditional video games, serious games act to convey meaningful information through interactive environments similar to real-life situations [11,12]. The use of serious games on computer and mobile phone platforms to promote awareness of health issues has increased in popularity over the past decade [13]. Technology is readily accessible in the United States, with 89% of households owning a computer or mobile phone device and 81% of households having an internet subscription [14]. Serious games teach specific skills or learning objectives and are created for educational purposes rather than entertainment [15].

Serious games have proven to be successful at educating users on various topics, including health, languages, computer science, mathematics, and geography [16]. Web game-based learning has been shown to positively affect user attitudes toward learning as well as increase the retention time of acquired knowledge [7]. Current serious games focus on specific disease states, making it difficult to generalize objectives to medication use [17]. Serious games have been reported to be desired for learning by patients. In one study, children picking up prescriptions in the pharmacy were reported to have asked for interactive games to learn about their medications [18].

Some existing systematic reviews examining the use of serious games include little information about medication use targeting specific disease states, such as diabetes [19,20], HIV prevention and care [21], asthma management [22,23], and epilepsy [24]. Other systematic reviews include broad search criteria, such as serious game use in health care [25], health care gamification [24], and serious games for young people living with long-term medical conditions [17].

### Objectives

The primary objective of this study was to assess the extent of serious games intended to educate patients about medication use and safety. In particular, this systematic review aimed to explore (1) serious games intended to educate patients about medication adherence, education, and safety; (2) types of theoretical frameworks used to develop serious games for medication use; and (3) sampling frames for evaluating serious games on medication use.

## Methods

### Search Strategy

A literature search was conducted using PubMed, Scopus, and Web of Science databases. The key terms included in the search were (serious game OR serious-games OR serious video-games OR serious games OR serious digital games OR serious electronic games OR serious gaming OR video game OR video-game) (drug OR drugs OR medication OR medications OR prescription OR prescriptions) (treatment OR therapy). Search results from each database were exported to Microsoft Excel, merged, and sorted for removal of duplicate citations.

### Study Selection

This review was conducted in accordance with the Preferred Reporting Items for Systematic Reviews and Meta-Analyses guidelines. Only original research articles were included in this systematic review. Initial screening of all abstracts and titles was conducted independently by SL and SB to determine whether to include or exclude an article based on selection criteria. Inclusion criteria were original research studies published in English for patients as end users involving a serious game, which focus on medication use and safety, addressing at least one of the following topics: (1) medication safety, (2) medication adherence, and (3) medication education. During the abstract and title screening phase, a level of agreement on inclusion and exclusion was achieved among the authors. A third author, HS, reconciled disagreements to achieve mutual consensus before moving to full-text review. Full-text articles were assessed for inclusion, and reasons were documented for all excluded papers.

Definitions of key terms are as follows:

- *Serious games*: A digital or computerized game designed for patients to increase their knowledge and awareness about medications or help them with medication use and safety.
- *Games for medication adherence*: Games that promote players to take medication at least as prescribed.
- *Games for medication education*: Games that teach players how medications work in the body or why the medication is an important component of patients’ treatment plan.
- *Games for medication safety*: Games about taking and handling medications in the proper way or safely to prevent medication errors.

### Data Extraction

A standard data extraction form was used to collect study authors, article title, year published, journal title, study design, brief description of methods, primary outcome measures, and conclusions by all the authors for the articles included for full-text inclusions in the last step. References of the papers initially found were not included for evaluation.

## Results

### Literature Overview

**Figure 1:**
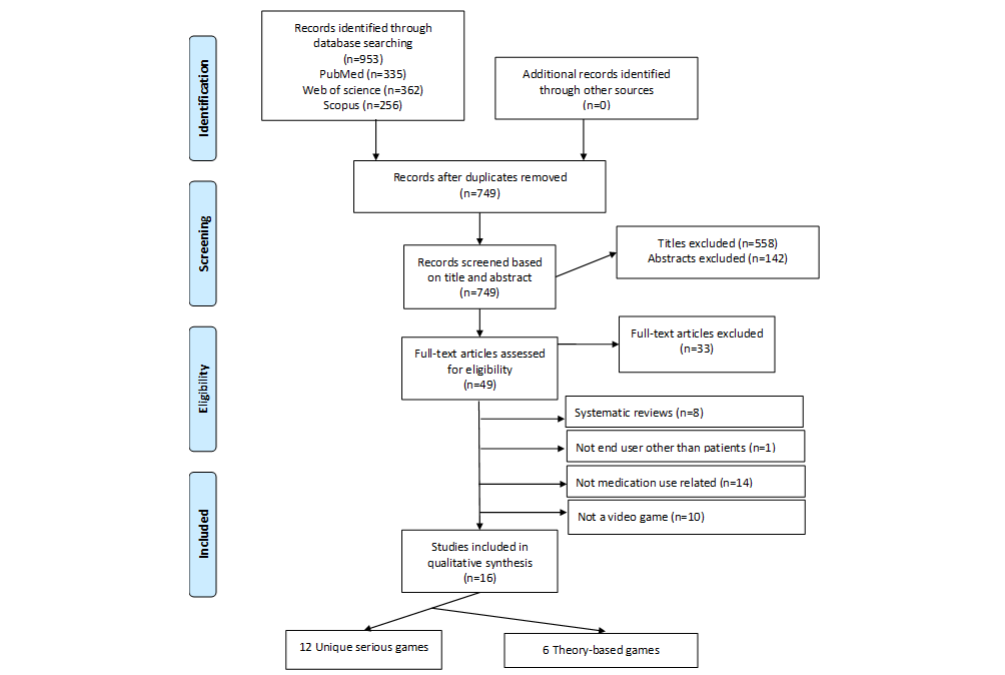
Preferred Reporting Items for Systematic Reviews and Meta-Analyses flow diagram of the included studies.

A total of 953 records were obtained after searching PubMed, Web of Science, and Scopus. After removing duplicates, 749 articles with unique titles were identified for title reviews. After title review, 558 studies were removed, and another 152 studies were removed after abstract review for not meeting the inclusion criteria. Studies were removed if they did not include a serious game focusing on patients as end users and based on medication use, adherence, and safety. A comprehensive review of 49 full-text articles was conducted, 33 of which were excluded. Systematic reviews, serious games for end users other than patients, nonmedication-related games, and nonvideo games were excluded (see Figure 1). As a result, 16 articles were included in this systematic review. The results are presented below based on the following three specific aims of the paper: (1) serious games intended to educate patients about medication adherence, education, and safety; (2) types of theoretical frameworks used to develop serious games for medication use; and (3) sampling frames for evaluating serious games on medication use.

A total of 16 articles on serious games published between 2003 and 2019 were found to incorporate medication use targeted toward patients. The 16 articles found focused on 12 unique serious games. Moreover, eight games were tested in adolescent populations, three games were tested in young adults, and one game was tested in adults only. In some cases, there were multiple articles focused on a single serious game. As shown in Multimedia Appendices 1 and 2, the included games could be grouped based on the disease state or education on a specific topic. All 12 games are described in Multimedia Appendices 1 and 2.

### Intention of the Serious Game

Most of these articles studied the change in patients’ knowledge about medications as an assessment of medication adherence. The following are examples of 8 games that assessed patients' understanding of medications for managing chronic and acute health conditions.

- Games for antiretroviral therapy (ART) and pre-exposure prophylaxis (PrEP) adherence: Viral Combat, Adherence Warrior, Epic Allies, and Battle Viro were developed to promote adherence to ART and PrEP [26-30]
- Game for cancer treatment medication adherence: Re-Mission [31]
- Game for diabetes medication education and adherence: L’Affaire Birmann [32,33]
- Game for asthma medication education: Wee Willie Wheezie [34]
- Games for microbiology and antibiotic education: Microbe Quest and e-Bug Junior and Senior [35-37]
- Game for prescription drug abuse education: CSI Web Adventures [38]
- Game for over-the-counter (OTC) medication safety: Alchemy Knights [39]

### Types of Theoretical Frameworks Used to Develop Serious Games for Medication Use

Of the 16 studies, seven used a theoretical model or framework in the game design process. The theories used in each game are mentioned in Multimedia Appendix 1. In *Viral Combat*, the information, motivation, and behavior (IMB) theory was used to promote medication behavior change [26]. *Adherence Warrior*, another game for HIV adherence, was guided by the social cognitive theory. Social cognitive theory goals included increased player self-efficacy, knowledge of HIV treatment goals, and social support [27]. *Battle Viro* was the only HIV-related game incorporating educational modules and did not report a specific theoretical framework or model that guided game development. In *Epic Allies*, the IMB model framework was used to develop objectives of increased ART adherence and social support. In *Re-Mission*, a game for cancer medication adherence, the behavioral objectives were developed based on the social cognitive theory and social learning theory [31]. The social cognitive theory was used to measure patient’s confidence in their ability to carry out adherence behaviors to reach a goal [31]. The social learning theory connects medication adherence to a social component such as a multiplayer serious game [31]. *L’Affaire Birman* was a serious game for type 1 diabetes education and did not use a theoretical framework. *Wee Willie Wheezie* had a single medication learning objective of proper inhaler use and used the precede-proceed model, which involves incorporating components such as predisposing, enabling, and reinforcing factors into game development [34]. One published study with an unnamed computer game used the pain and medication model to teach players of any age how to manage activities of daily living through balancing activity level with the quantity of medication taken postoperatively [40]. The theoretical framework used in development was a blend of the middle-range theory of chronic illness, adult learning theory, and the experiential learning theory. The middle-range theory of chronic illness involves reflection and decision making, such as choosing when to take pain medication [40]. The adult learning theory incorporates self-directed learning methods, which is a preferred learning method for adults [40]. The experiential learning theory involves learning through observation, abstract conceptualization, and experimentation [40]. *CSI Web Adventures* used the theory of reasoned action to display the negative consequences of abusing prescription drugs, such as opioids in two separate case studies [38].

### Sampling Frames Primary Results

*Viral Combat*, *Adherence Warrior*, *Epic Allies*, and *Battle Viro* were developed to promote adherence to ART and PrEP [26-30]. The target audience was aged between 13 and 35 years in all HIV-focused games [8,27-30]. Each game used three to four modalities to achieve learning objectives, summarized in Multimedia Appendices 1 and 2.

*Viral Combat*, an iPhone gaming app published only through the game development phase, incorporated a *Smart Pill Bottle Cap* and text messages to patients aged 18 to 35 years to promote adherence to PrEP [26]. A *Smart Pill Bottle Cap* reports to the app when the medication bottle is opened [26]. A small trial of nine participants showed an 88% satisfaction toward the game, and 100% of the participants would recommend it to a friend [30].

*Adherence Warrior,* a mobile gaming app for patients aged 13 to 24 years, promoted adherence while maintaining player privacy. Text messages were sent to patients to promote ART adherence [27]. A mixed method study had 12 participants having rank level of agreement on a scale of 1 to 5, with 5 being strong agreement to game characteristics. The study reported a median score of 5 of having fun while playing (*P*=.03), and players preferred to play games about topics other than the immune system (*P*=.01) [27]. No statistical significance was found to support whether participants would play the game if it were available or if they would use the game to take HIV medications [27].

*Battle Viro*, an iPhone gaming app targeted to patients aged 14 to 26 years, incorporated a *Smart Pill Bottle Cap*, text messages, and educational modules. Learning outcomes were to improve ART adherence, increase social support, increase HIV- and ART-related knowledge, and visualize progress. In a randomized controlled trial of 61 participants starting with a detectable viral load, the experimental condition had 23% greater adherence (*P*=.05) compared with the control group and a 0.96 log greater decrease in viral load (*P*=.04) [8,30].

*Epic Allies* was the only HIV medication adherence mobile gaming app that did not incorporate an electronic pill bottle or text messages. The target age for this game was 16 to 29 years [28,29]. The distinguishing modality of *Epic Allies* was a dashboard displaying various lifestyle behaviors, such as smoking, medication adherence, and mood [28]. A sample size of 20 study participants through focus groups supported game acceptability [28,29].

*Re-Mission* focused on increasing oral chemotherapy adherence and strategizing the use of medications to treat the side effects of oral chemotherapy for people aged 12 to 29 years [31]. Players control a robot, *Roxxi*, with the goal of adhering to oral chemotherapy and combating negative effects through taking medications such as stool softeners and antibiotics [31]. A randomized control trial of 375 participants yielded no significant results for adherence but a 9.8% increase in Trimethoprim and Sulfamethoxazole adherence (*P*=.01) [31] and a significant increase in player’s self-efficacy for medication adherence (*P*=.01) [31].

Two studies on one game specifically included a diabetic medication-centered learning objective [28,29]. Target ages in published studies ranged from 10 to 19 years [32,33]. *L’Affaire Birman* was a serious game targeted toward children living with type 1 diabetes [32]. Players used a strategic approach to adjust the game character’s insulin based on lifestyle factors such as food intake, physical activity, and glucose levels. With no results or sample characteristics reported, the authors suggested that further testing is needed to assess the effectiveness in the clinical setting [32].

*Wee Willie Wheezie*, a 3-level computer-assisted instruction program targeted toward children aged 7 to 12 years, had a single medication learning objective of proper inhaler use [34]. The players chose the correct medication to avoid asthma symptoms, exacerbations, and hospital trips [34]. A randomized controlled trial of 148 participants found no significant improvement in player’s asthma symptoms or quality of life parameters [34].

In an unnamed game, patients learned safe medication regimens by using the icons in the game to learn about the side effects of each medication. [40]. An evaluation study of 20 participants aged 24 to 67 years found an increase in knowledge on strategies to manage pain (*P*<.001) [40].

*e-Bug* Junior and Senior were multiple-module educational adventure computer games targeted toward students ranging in age from 9 to 12 years and 13 to 15 years, respectively [35,36]. Each game had one module teaching the purpose of antibiotics and the importance of taking the full course. An evaluation study of 129 students yielded 98% positive comments about the senior game and no efficacy results [35,36].

*Microbe Quest* was a mobile gaming app targeted to patients aged 9 to 12 years [37]. A single level of gameplay introduced the concept of antibiotic resistance as a consequence of not finishing a full course of antibiotics [37]. No statistically significant results in learning objective retention were found in the initial pilot study of 19 participants [37].

*CSI Web Adventures* simulated a prescription drug abuse crime scene and took players through the science of forensic analysis [38]. This computer game was targeted toward people aged 14 to 18 years. A sample of 179 players conveyed negative attitudes toward illegal crimes in the baseline and game testing phases [38]. CSI Web Adventures is reported to need more testing before significant results can contribute to specific opioid safety-related learning objectives [38].

*Alchemy Knights,* a serious game available on the Web, was geared toward ages 9 to 12 years. The game taught players about responsible OTC medication safety, drug-drug interactions, and the consequences of misusing medications [39]. A pilot study of nine participants showed 78% increased knowledge in medication safety from a pretest to posttest analysis [39]. Results will be used to improve the game for future use [39].

## Discussion

### Overview

This systematic review offers valuable additions to the current evidence-based literature by examining serious games for patients that incorporate medication adherence, education, and safety. Existing systematic reviews explore serious games for health focused on the management of specific disease states, such as HIV, diabetes, and asthma, and on the positive impact of serious game education in each respective disease state [19-24]. Although other reviews target broad topics such as health care gamification and serious games to educate health care workers [7,17,25], no reviews focus solely on examined medication use [7,15-17,19,20]. The identified serious games that incorporate the use of medications are mainly focused on improving adherence, whereas medication safety is not widely explored. In addition, there is a lack of research on the efficacy and usability of medication-focused serious games often because of small and nonrepresentative sample sizes, which limit the generalizability of existing studies. Very few serious games described how theoretical frameworks were incorporated during development, showing an area for improvement in literature [26,28,34]. This systematic review signifies the need for the creation of serious games focused on medication adherence, education, safety, testing of existing serious games for efficacy and effectiveness, an evidence-based theory-driven approach for serious game design, and large-scale testing with randomized samples to improve generalizability.

### Medication Incorporation

Each serious game included in this review was analyzed for the extent and quality of medication-related topics. The included games had a medication-related learning objective incorporated into a gameplay feature. Of the 12 unique serious games included in this study, most did not have medication as a principal component. The sole content in *Re-Mission*, *Alchemy Knights*, and the unnamed pain management game were medication adherence, education, and safety, respectively [31,39,40]. ART and PrEP adherence was incorporated in *Viral Combat, Adherence Warrior, Epic Allies, and Battle Viro* through social support, text reminders, computerized pill bottles, and various point incentives [8,26-30]. *Microbe Quest, e-Bug, Wee Willie Wheezie, and CSI Web Adventures* included a single medication-related module [34-38]. Although medication is not extensively incorporated into serious games, this demonstrates a modality of patient education to be explored in the future.

### Theoretical Frameworks

Validated social, behavioral, and game theories such as IMB theory, social cognitive theory, precede-proceed model, middle-range theory of chronic illness, adult learning theory, experiential learning theory, and the theory of reasoned action as included in this review are developed and defined iteratively over time. Using these theories for game development and testing can improve the effectiveness of these serious games [26,30]. On the evaluation of the 12 serious games identified in this study, six incorporated varying degrees of theoretical frameworks to support game development and testing. A total of three articles about two serious games extensively focused on IMB theory for game development [26,28,29]. The goal of the IMB model in *Epic Allies* and *Battle Vivo* was to change specific health-related behaviors such as medication adherence through a combination of health education, self-motivation, and gaining required skills [26,28,29]. Game mechanics in *Epic Allies* were designed to motivate ART adherence in young men who have sex with men and long-term game use [28,29]. Another article extensively described the use of the precede-proceed model in game design of *Wee Willie Wheezie* [34]. Two games mentioned the social cognitive theory, and one game mentioned the theory of reasoned action, but neither of them described any specific details about using and integrating the theory in game design and mechanics [8,27,30,38]. Future goals in *Alchemy Knights* indicate examining theoretical contributions for further game development [39]. Although most of the serious games involving theory in their design used small sample sizes for testing their efficacy and effectiveness, positive outcomes were still demonstrated by a few [26,28,38]. A common theme identified was a lack of statistical power to test the efficacy of theory outcomes because of the small sample size or result usability [8,26-30,34,39,40]. Future literature describing serious game development should incorporate more thorough descriptions of the theoretical frameworks used and larger sample sizes.

### Medication Adherence, Education, and Safety

Of the 12 serious games included in this study, six focused on improving medication adherence [7,8,26-31,38,40], three targeted medication education [29,32], and three were aimed at providing medication safety [35-39]. Medication nonadherence is a prominent issue in health care, which leads to increased costs and comorbidities. By using innovative approaches to teach patients the value of adhering to medication, serious games can assist in improving medication therapy outcomes. Serious games educating users on medication misuse or promoting safe usage of medication were lacking in the literature. Although the authors recognize that there is a thin line between medication safety and education, there were many more games with education and adherence components when compared with medication safety components such as preventing inappropriate use [36-40]. Future serious games with medication use as a component should incorporate learning objectives targeting medication safety principles to prevent adverse drug events.

### Sampling Frames

This study recognizes that the majority of serious games included in this study were tested for usability and functionality with very small sample sizes. Only five of the included studies had more than 100 participants [29,31,34,38]. These small sample sizes limit the external validity of these studies, thus reducing the generalizability of the results to larger populations. In the future, the effectiveness of these serious games must be assessed using larger sample sizes to investigate their impact on patient’s knowledge and understanding about medication adherence, education, and safety.

Regarding geographical distribution of samples used in the studies, only one study included patients from outside of a single state [31], whereas other articles used convenient samples from a single clinic, city, or state [8,26,27,29,30,32,34-38]. By limiting the participants to a single site, geographical location or a specific age group, the external validity of the results from these games is again compromised. Of the 12 serious games, eight were tested in adolescents, three were tested in young adults, and one was tested in adults. Although adolescents benefit from serious games, adults could also benefit from serious games and should be a future area of exploration in serious game usability studies. In the future, more studies with randomized and diverse populations could increase the statistical power of these results.

The published literature on medication-based serious games has a strong focus on game design, mechanics, and methodologies rather than the effectiveness of the game. Of the included articles, seven focused on game design and the plans for future game development without elaboration on outcomes and game efficacy [8,27,29,30,33-36]. A total of four studies focused on the intended outcomes of the games and whether they were met [26,31,37]. The common method of evaluation was surveys on gameplayers’ satisfaction while playing but not the information that they sustained and retained from the games. Quantifying whether learning objectives were met is necessary to determine the success of serious games for future studies. Thus, future serious game design development should include plans for rigorous testing of efficacy.

### Limitations

The authors recognize key limitations of this study. First, only 3 databases were used for the literature search. Although the extent of duplicates supported a thorough search, there is a chance that there are relevant papers that were not included. Second, only papers written in English were included. This may have excluded papers from non-English–speaking countries. Finally, small sample sizes were used to test most games included in this study. None of the literature discussed the sustainability of the games post study, and most of the included games did not have long-standing follow-up data on their participants.

### Conclusions

There have been limited studies on serious games for health that incorporate medication use. The findings from these studies focus on developing and testing serious games that teach patients about medication use and safety. Most of these studies do not apply a theoretical framework in the design and assessment of these games. The development of serious games for patient medication use, education, and adherence should incorporate evidence-based and theory-driven methods to ensure maximum retention of the learning objectives by study participants and game players. More diverse, randomized studies with long-term data collection need to be conducted to demonstrate the effectiveness of serious games in this area. Serious games have the potential to reduce patients’ knowledge gaps and address misconceptions, which may lead to improved medication adherence and reduced errors. This review shows that there has been an increased interest in the application of serious games to improve medication use outcomes, and it is expected that this review will help advance the effectiveness of game development in the future.

## Appendix

Multimedia Appendix 1 Table 1. Inclusion of medication-focused serious games.

Multimedia Appendix 2 Table 2. Summary of learning objectives and outcomes of serious games.

## Abbreviations

ART: antiretroviral therapy
IMB: information, motivation, and behavior
NIH: National Institutes of Health
OTC: over-the-counter
PrEP: pre-exposure prophylaxis
PRISMA: Preferred Reporting Items for Systematic Reviews and Meta-Analyses
UW: University of Wisconsin

## References

[1] Brown MT, Bussell J, Dutta S, Davis K, Strong S, Mathew S (2016). Medication adherence: truth and consequences. Am J Med Sci.

[2] Hodges NL, Spiller HA, Casavant MJ, Chounthirath T, Smith GA (2018). Non-health care facility medication errors resulting in serious medical outcomes. Clin Toxicol (Phila).

[3] Chan DC, Shrank WH, Cutler D, Jan S, Fischer MA, Liu J, Avorn J, Solomon D, Brookhart MA, Choudhry NK (2010). Patient, physician, and payment predictors of statin adherence. Med Care.

[4] (2009). US Food & Drug Administration.

[5] Iuga AO, McGuire MJ (2014). Adherence and health care costs. Risk Manag Healthc Policy.

[6] Vivolo-Kantor AM, Seth P, Gladden RM, Mattson CL, Baldwin GT, Kite-Powell A, Coletta MA (2018). Vital Signs: trends in emergency department visits for suspected opioid overdoses - United States, July 2016-September 2017. MMWR Morb Mortal Wkly Rep.

[7] Pereira P, Duarte E, Rebelo F, Noriega P, Marcus A (2014). A review of gamification for health-related contexts. Design, User Experience, and Usability. User Experience Design for Diverse Interaction Platforms and Environments.

[8] Whiteley L, Brown L, Lally M, Heck N, van den Berg JJ (2018). A mobile gaming intervention to increase adherence to antiretroviral treatment for youth living with HIV: Development guided by the information, motivation, and behavioral skills model. JMIR Mhealth Uhealth.

[9] Girard C, Ecalle J, Magnan A (2013). Serious games as new educational tools: how effective are they? A meta-analysis of recent studies. J Comput Assist Learn.

[10] Bruggers CS, Altizer RA, Kessler RR, Caldwell CB, Coppersmith K, Warner L, Davies B, Paterson W, Wilcken J, D'Ambrosio TA, German ML, Hanson GR, Gershan LA, Korenberg JR, Bulaj G (2012). Patient-empowerment interactive technologies. Sci Transl Med.

[11] Susi T, Johannesson M, Backlund P (2007). DiVA.

[12] Marsh T (2011). Serious games continuum: Between games for purpose and experiential environments for purpose. Entertain Comput.

[13] Klasnja P, Pratt W (2012). Healthcare in the pocket: mapping the space of mobile-phone health interventions. J Biomed Inform.

[14] Villanti AC, Johnson AL, Ilakkuvan V, Jacobs MA, Graham AL, Rath JM (2017). Social media use and access to digital technology in US young adults in 2016. J Med Internet Res.

[15] Olszewski AE, Wolbrink TA (2017). Serious gaming in medical education: a proposed structured framework for game development. Simul Healthc.

[16] Rodriguez DM, Teesson M, Newton NC (2014). A systematic review of computerised serious educational games about alcohol and other drugs for adolescents. Drug Alcohol Rev.

[17] Wilson AS, McDonagh JE (2011). Moving on: use of computer games during transitional care for young people with long term medical conditions. Proceedings of the 6th european conference on games based learning.

[18] Abraham O, Brothers A, Alexander DS, Carpenter DM (2017). Pediatric medication use experiences and patient counseling in community pharmacies: Perspectives of children and parents. J Am Pharm Assoc (2003).

[19] Theng YL, Lee JW, Patinadan PV, Foo SS (2015). The use of videogames, gamification, and virtual environments in the self-management of diabetes: a systematic review of evidence. Games Health J.

[20] DeShazo J, Harris L, Pratt W (2010). Effective intervention or child's play? A review of video games for diabetes education. Diabetes Technol Ther.

[21] Hightow-Weidman LB, Muessig KE, Bauermeister JA, LeGrand S, Fiellin LE (2017). The future of digital games for HIV prevention and care. Curr Opin HIV AIDS.

[22] Drummond D, Monnier D, Tesnière A, Hadchouel A (2017). A systematic review of serious games in asthma education. Pediatr Allergy Immunol.

[23] Baptist AP, Islam N, Joseph CL (2016). Technology-based interventions for asthma-can they help decrease health disparities?. J Allergy Clin Immunol Pract.

[24] Rahim MI, Thomas RH (2017). Gamification of medication adherence in epilepsy. Seizure.

[25] Kearns C (2015). Prescription play: A primer on innovative use of video games technology in healthcare. J Vis Commun Med.

[26] Whiteley L, Mena L, Craker LK, Healy MG, Brown LK (2019). Creating a theoretically grounded gaming app to increase adherence to pre-exposure prophylaxis: lessons from the development of the viral combat mobile phone game. JMIR Serious Games.

[27] Castel AD, Qasmieh S, Greenberg D, Ellenberger N, Howell TH, Griffith C, Wilbourn BC, Ganesan K, Hussein N, Ralte G, Rakhmanina N (2018). Digital gaming to improve adherence among adolescents and young adults living with HIV: Mixed-Methods study to test feasibility and acceptability. JMIR Serious Games.

[28] LeGrand S, Muessig KE, McNulty T, Soni K, Knudtson K, Lemann A, Nwoko N, Hightow-Weidman LB (2016). Epic Allies: development of a gaming app to improve antiretroviral therapy adherence among young HIV-positive men who have sex with men. JMIR Serious Games.

[29] LeGrand S, Muessig KE, Platt A, Soni K, Egger JR, Nwoko N, McNulty T, Hightow-Weidman LB (2018). Epic Allies, a gamified mobile phone app to improve engagement in care, antiretroviral uptake, and adherence among young men who have sex with men and young transgender women who have sex with men: protocol for a randomized controlled trial. JMIR Res Protoc.

[30] Whiteley L, Brown LK, Mena L, Craker L, Arnold T (2018). Enhancing health among youth living with HIV using an iPhone game. AIDS Care.

[31] Kato PM, Cole SW, Bradlyn AS, Pollock BH (2008). A video game improves behavioral outcomes in adolescents and young adults with cancer: a randomized trial. Pediatrics.

[32] Joubert M, Armand C, Morera J, Tokayeva L, Guillaume A, Reznik Y (2016). Impact of a serious videogame designed for flexible insulin therapy on the knowledge and behaviors of children with type 1 diabetes: The Ludidiab Pilot Study. Diabetes Technol Ther.

[33] Friess R, Kolas N, Knoch J (2014). Game Design of a Health Game for Supporting the Compliance of Adolescents With Diabetes. https://link.springer.com/content/pdf/10.1007/978-3-658-07141-7.pdf#page=46.

[34] Huss K, Winkelstein M, Nanda J, Naumann PL, Sloand ED, Huss RW (2003). Computer game for inner-city children does not improve asthma outcomes. J Pediatr Health Care.

[35] Lazareck LJ, Farrell D, Kostkova P, Lecky DM, McNulty CA, Weerasinghe D (2010). Learning by gaming - evaluation of an online game for children. Conf Proc IEEE Eng Med Biol Soc.

[36] Farrell D, Kostkova P, Lazareck L, Weerasinghe D, Weinberg J, Lecky DM, Adriaenssens N, Herotová TK, Holt J, Touboul P, Merakou K, Koncan R, Olczak-Pienkowska A, Avô AB, Campos J, McNulty CA (2011). Developing e-Bug web games to teach microbiology. J Antimicrob Chemother.

[37] Molnar A, Kostkova P (2018). Learning about Hygiene and Antibiotic Resistance through Mobile Games: Evaluation of Learning Effectiveness. Proceedings of the 2018 International Conference on Digital Health.

[38] Klisch Y, Bowling KG, Miller LM, Ramos MA (2013). The impact of science education games on prescription drug abuse attitudes among teens: a case study. J Drug Educ.

[39] Abraham O, Feathers A, Grieve L, Babichenko D (2019). Developing and piloting a serious game to educate children about over‐the‐counter medication safety. J Pharm Health Serv Res.

[40] Ingadottir B, Blondal K, Thue D, Zoega S, Thylen I, Jaarsma T (2017). Development, usability, and efficacy of a serious game to help patients learn about pain management after surgery: an evaluation study. JMIR Serious Games.

