# Supplementary material for: Investigating Serious Games That Incorporate Medication Use for Patients: Systematic Literature Review
Source: JMIR Serious Games. 2020 Apr 29;8(2):e16096. doi: 10.2196/16096 (PMC7221639; doi:10.2196/16096)
Supplement: Multimedia Appendix 1 [file games_v8i2e16096_app1.docx]

**Table 1.** Inclusion of medication-focused serious games (n=16).

| Study | Game | Age (years) | | Development stage | Components | Theory |
| --- | --- | --- | --- | --- | --- | --- |
| Whiteley et al [26] | Viral Combat | | 18-35 | Game development | Reward system, educational content action adventures, and text messages | IMB^a^ |
| Castel et al [27] | Adherence Warrior | | 13-24 | Mixed method study | Wisepill technology, action adventures, reward system, text messages, and provider connection | Social cognitive |
| LeGrand et al [28] | Epic Allies | | 18-29 | Game development and focus groups | Medication reminder, modules on ART^b^ or HIV, action adventures, and lifestyle dashboard | IMB |
| LeGrand et al [29] | Epic Allies | | 16-24 | Randomized controlled trial | Medication reminder, modules on ART or HIV, action adventures, lifestyle, and dashboard | IMB |
| Whiteley et al [8] | Battle Viro | | 18-26 | Game development | Smart pill cap, text messages, and action game | None |
| Whiteley et al [30] | Battle Viro | | 14-26 | Controlled trial | Smart pill cap, text messages, and action game | None |
| Joubert et al [32] | L’Affaire Birman | | 11-18 | Game development | Problem solving and strategy based | None |
| Friess et al [33] | No name | | 10-19 | Pregame development | Serious video game | None |
| Ingadottir et al [35] | No name | | 24-67 | Evaluation study | Avatar-based game and medication board | Middle-range theory, adult learning, and experiential learning |
| Lazareck et al [36] | e-Bug (Sr) | | 13-15 | Senior game and evaluation study | Adventure-based game | None |
| Farrell et al [37] | e-Bug Junior  e-Bug Senior | | 9-12  13-15 | Game development | Junior: 5 educational modules  Senior: story-based adventure game | None |
| Molnar et al [38] | Microbe Quest | | 9-12 | Game development pilot study | Adventure game and pre- and postgame quiz | None |
| Kato et al [31] | Re-mission | | 12-29 | Randomized controlled trial | Simulation video game | Social cognitive and social learning |
| Huss et al [34] | Wee Willie Wheezie | | 7-12 | Randomized controlled trial | Three-level computer-assisted instruction program | Precede-proceed |
| Klisch et al [39] | CSI Web Adventures: Cases 4 and 5 | | 14-18 | Case study | Forensic role-play case study | Reasoned action |
| Abraham et al [40] | Alchemy Knights | | 9-12 | Game development pilot study | Serious video game | None |

^a^IMB: information, motivation, and behavior. ^b^ART: antiretroviral therapy.
