# Supplementary material for: Investigating Serious Games That Incorporate Medication Use for Patients: Systematic Literature Review
Source: JMIR Serious Games. 2020 Apr 29;8(2):e16096. doi: 10.2196/16096 (PMC7221639; doi:10.2196/16096)
Supplement: Multimedia Appendix 2 [file games_v8i2e16096_app2.docx]

**Table 2.** Summary of learning objectives and outcomes of serious games (n=16).

| Game | Learning objectives | Study outcomes and results |
| --- | --- | --- |
| Viral Combat [26] | - Adherence to pre-exposure prophylaxis as prescribed - Clinician interaction skills - Practice safe sex | - Client service questionnaire and session evaluation form scores of 9 showed 88% game satisfaction - Need randomized trial to test adherence behavior |
| Adherence Warrior [27] | - Increase adherence through incentivized in-game energy boosts - Maintain player privacy | - Long-term feasibility and acceptability found in mixed method study of Wisepill dispenser and video game |
| Epic Allies [28,29] | - Information, Motivation, and Behavior model framework as a framework for medication adherence and social support - Visual board to increase visibility of adherence patterns | - Focus groups showed game acceptability - Need randomized trial for further results |
| Battle Viro [8,30] | - Increase adherence and motivation to seek treatment - Build social support and efficacy through education on ART^a^ | - Controlled trial showed improvements in ART and HIV knowledge - Participants saw improved social support - Improved ART adherence was impactful in decreasing viral load |
| L’Affaire Birman [32,33] | - Manage character’s diabetes through adjusting insulin dose based on food intake, physical activity, and glucose level | - Game slightly improves functional insulin therapy knowledge - Further studies needed to test efficacy in clinical setting |
| No Name [35] | - Manage daily activities of living - ·Manage postoperative pain | - Statistically significant increase in knowledge of strategies to improve pain management after playing (*P<*.001) |
| e-Bug (Junior game) [36] | - Sometimes it is necessary to use antibiotics - It is important to take the full course of antibiotics | - No results posted |
| e-Bug (Senior game) [36,37] | - Learn negative outcomes of not finishing antibiotic course and side effects of using someone else’s antibiotics | - In focus groups, 98% reported positive comments about the game |
| Microbe Quest [38] | - Antibiotics harm good and bad bacteria - Antibiotics are used to fight bacterial infections - If prescribed, the full course of antibiotics must be used - When antibiotics are used incorrectly, bacteria develop resistance | Learning across all learning objectives was not statistically significant   - Hypotheses could not be confirmed by small sample size |
| Re-Mission [31] | - Destroy cancer cells - Use medications to combat side effects of chemotherapy - Improve oral chemotherapy adherence | - Randomized controlled trial showed increased medication adherence in patients with oral chemotherapy - Intervention group showed increased knowledge of cancer therapy and increased self-efficacy |
| Wee Willie Wheezie [34] | - Select correct medications to avoid asthma symptoms, exacerbations, and hospital trips | - Randomized controlled trial showed no significant improvements in asthma symptoms and quality of life parameters |
| Alchemy Knights [40] | - Learn over-the-counter medication safety - Learn about drug-drug interactions and consequences of medication build up - Choose the correct option and dose to stay alive in the game | - Increase in medication safety knowledge in some questions post the game - Results will be used to refine game and used toward other games |

^a^ART: antiretroviral therapy.
